# Effect of a Discharge Checklist in the Emergency Department on Physicians' Satisfaction, Time Management, and Confidence: A Cross-Sectional Study

**DOI:** 10.7759/cureus.81842

**Published:** 2025-04-07

**Authors:** Maan Jamjoom, Rand A Maddah, Shahad Kamal, Bsaim A Altirkistani, Nada A Alhazmi, Hussa A Alrashid, Zyad E Raffah, Razzan A Altirkistani

**Affiliations:** 1 Emergency Medicine, King Abdullah Medical City, Jeddah, SAU; 2 Emergency Medicine, King Abdulaziz Medical City, Ministry of National Guard, Jeddah, SAU; 3 College of Medicine, King Saud bin Abdulaziz University for Health Sciences, Jeddah, SAU; 4 Emergency Medicine, King Abdullah International Medical Research Center, Jeddah, SAU; 5 Nursing, King Abdullah Medical City, Makkah, SAU

**Keywords:** checklist, discharge, emergency medicine, physicians, satisfaction

## Abstract

Introduction

Timely and accurate discharge communication is important in continuing patient care. Discharge instructions are often rushed, and patients frequently do not understand aspects of their discharge, particularly medication management. This study aimed to assess the effect of an evidence-based discharge checklist on physicians and residents by measuring their satisfaction with the current discharge process versus the results after applying a discharge checklist.

Subjects and methods

This cross-sectional study compared physicians' satisfaction and confidence levels before and after the discharge checklist was administered. The physicians filled out a self-administered questionnaire electronically consisting of demographic data (i.e., gender, main specialty, professional rank) and a 5-point Likert scale questionnaire to assess the level of satisfaction, confidence, and discharge skills.

Results

A total of 38 physicians participated in this study. Male physicians constituted the majority of the respondents (76.3%). There were no significant differences between the level of satisfaction and confidence before and after the use of the discharge checklist. However, physicians (staff physicians, fellows, and consultants) tended to agree that discharge skills are time-consuming, whereas residents were more likely to agree that the discharge checklist increased their confidence levels. Interestingly, dissatisfaction with the discharge checklist was prevalent among emergency residents.

Conclusion

No evidence suggests that physician satisfaction improved after the use of the discharge checklist. Emergency department physicians tended to exhibit dissatisfaction after they used the checklist. However, resident physicians may exhibit better confidence in new discharge methods. Prospective studies may help us measure the effects of discharge checklists over time.

## Introduction

Timely and accurate discharge communication is important in continuing patient care. Delivery of discharge instructions is often rushed, and patients frequently do not understand aspects of their discharge, particularly medication management [1,2]. Patients who have poor comprehension of discharge instructions from the emergency department (ED) may have higher rates of ED visits, hospital readmissions [3-5], and medication errors [6]. They also lack knowledge about their diagnosis, follow-up care, and treatment. Therefore, improving patients’ understanding is likely to improve health outcomes and avoid unnecessary healthcare utilization and costs [7,8]. Effective discharge summaries reduce adverse drug events, unplanned hospital readmission, post-discharge complications, and mortality, and increase patient and care satisfaction [9-11]. In 2005, a prospective randomized controlled clinical trial showed significant improvements in discharge planning involvement, health service access, confidence within discharge procedures, and opinion of discharge based on previous experience in patients who received the discharge care plan [12]. Additionally, a 2015 study in which a checklist was used improved physicians’ perceived confidence in discharge, reminded them to complete necessary discharge tasks, and increased the perceived efficiency of the discharge process [13]. Studies have shown that the majority of general practitioners’ satisfaction with the quality and timeliness of discharge summaries also improved with the use of the electronic discharge summary [14-16]. In a study from 2011 to 2013, the impact of discharge instructions on a patient’s experience after the implementation of their revised discharge instructions was measured, and patient satisfaction significantly improved. Patients responded that they felt ready for discharge and were satisfied with instructions for home care [17]. Therefore, this study aims to assess and measure the effect of an evidence-based discharge checklist on physicians’ satisfaction at King Abdulaziz Medical City, Jeddah, Kingdom of Saudi Arabia.

## Materials and methods

**Figure 1:**
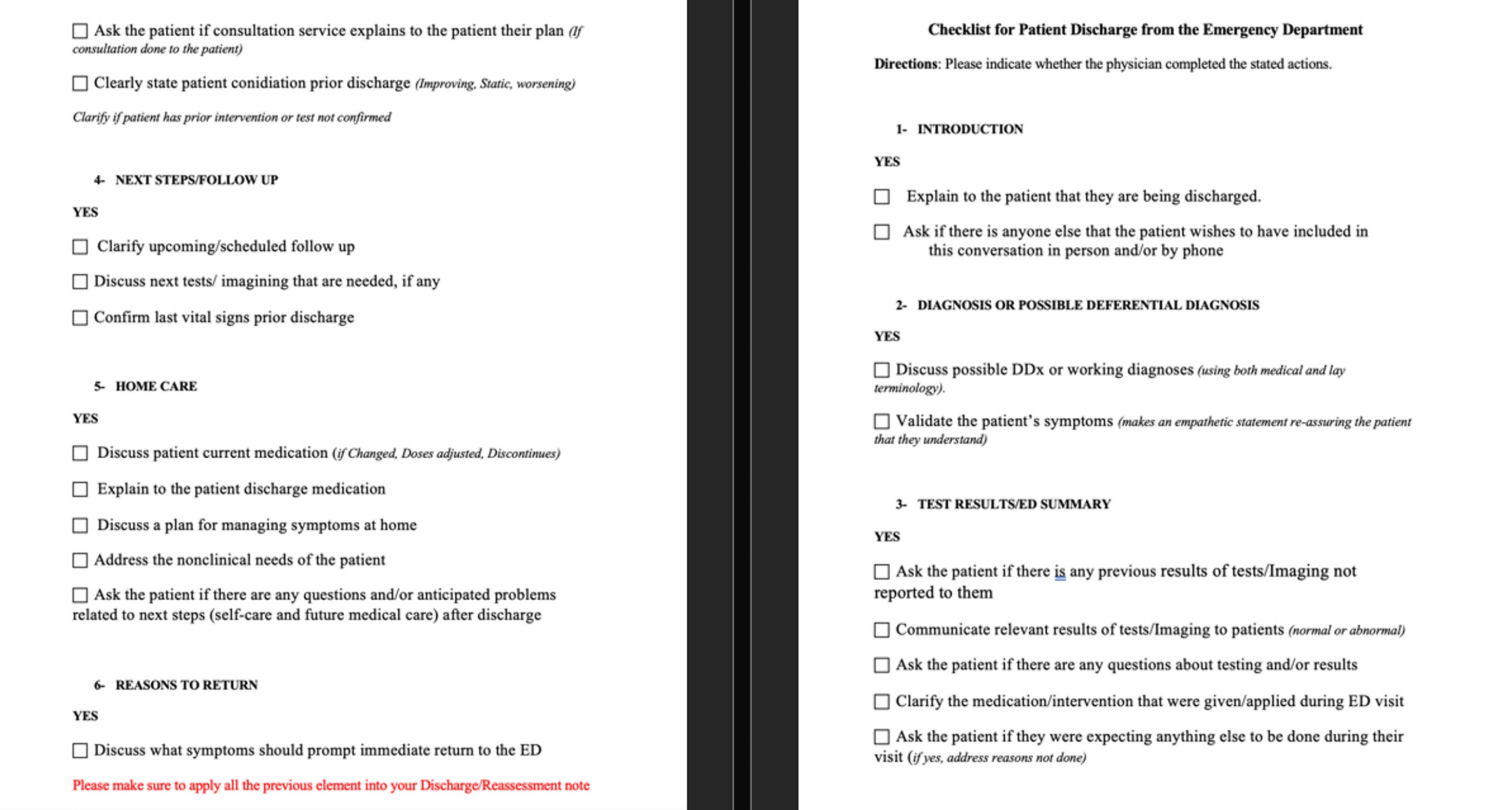
Checklist Checklist made by Dr. Maan Jamjoom

The study has a prospective cross-sectional design, as it aimed to assess the effect of an evidence-based discharge checklist on physicians by measuring their satisfaction with the discharge process via an electronic survey that was designed to contain questions with measurable outcomes distributed to participants before and after checklist implantation (Figure 1). The study was conducted in a single center at King Abdulaziz Medical City. The inclusion criterion included physicians (emergency medicine, internal medicine, pediatric, oncology, general surgery, family medicine) who are eligible to discharge patients from the ED as part of their progress/consultation notes. Before applying the checklist to physicians, they were given an electronic structured questionnaire that assessed the current satisfaction of physicians with their confidence with respect to the current discharge process of patients. Three months after the checklist was implemented, from November 5, 2023, until February 5, 2024, the same physicians were reached, and an electronic structured questionnaire assessing their satisfaction with the newly applied checklists was given again to determine the effectiveness of the checklist. After the physiotherapists completed the follow-up questionnaire, the data were extracted from the survey and entered into a Microsoft Excel spreadsheet for aggregation. Eventually, the data were exported for data analysis. The elements of the questionnaire consisted of demographic information, questions related to the discharge process, and their statements. The following question was used to assess satisfaction after discharge checklist use: "How satisfied are you with the new discharge checklist in terms of data completion and documentation?" Categories such as "strongly dissatisfied," "dissatisfied," and "neutral" were combined and classified as "dissatisfied," whereas categories such as "satisfied," and "strongly satisfied" were combined and classified as "satisfied." The confidence level was assessed by asking the following: "How would you rate your confidence level in discharging patients from the ER with the new discharge checklist?" Similar criteria were applied to classify the confidence levels, such as "very unconfident,” “unconfident,” “neutral" being combined and named "not confident," whereas categories "confident” and “very confident" were combined and named "confident."

Statistical analysis

Descriptive statistics are presented as numbers and percentages (%) for all categorical variables. The comparison of satisfaction and confidence levels before and after discharge was conducted using the Wilcoxon signed-rank test. Additionally, the satisfaction levels of physicians and residents before and after using the discharge checklist were measured using Fisher’s exact test. In addition, the relationship between the demographic data and the satisfaction levels after the discharge checklist was conducted using the Fischer exact test. P-values of less than 0.05 were considered significant. The data were analyzed using the software program Statistical Packages for Software Sciences (SPSS) version 26 (IBM Corp., Armonk, NY, USA).

## Results

A total of 38 physicians completed the survey. As shown in Table 1, the majority were male physicians (76.3%). Emergency medicine (57.9%) was the most prominent specialty, while the most common professional rank was resident postgraduate year (PGY) 2 (31.6%).

**Table 1:** Basic demographic characteristics of the physicians (n=38) PGY, postgraduate year

Study variables	N (%)
Gender	
Male	29 (76.3%)
Female	09 (23.7%)
Main specialty	
Emergency medicine	22 (57.9%^)
Internal medicine	03 (07.9%)
Pediatrics	07 (18.4%)
General Surgery	04 (10.5%)
Oncology	01 (02.6%)
Family medicine	01 (02.6%)
Professional rank	
Resident PGY1	0
Resident PGY2	12 (31.6%)
Resident PGY3	08 (21.1%)
Resident PGY4	07 (18.4%)
Staff physician	04 (10.5%)
Specialist/fellow	05 (13.2%)
Consultant	02 (05.3%)

When the satisfaction and confidence levels of physicians before and after the discharge checklist was applied (Table 2) were compared, the differences in satisfaction, confidence, discharge skills, and discharge checklist effects on confidence before and after the checklist was applied were not statistically significant (p>0.05).

**Table 2:** Comparison of physician satisfaction and confidence before and after the discharge checklist was administered (n=38) ^§^P-values were calculated using the Wilcoxon signed-rank test.

Satisfaction items	Before, N (%)	After, N (%)	P-value^§^
Level of satisfaction with the discharge process			
Very dissatisfied	02 (05.3%)	01 (02.6%)	0.762
Dissatisfied	02 (05.3%)	01 (02.6%)	
Neutral	08 (21.1%)	12 (31.6%)	
Satisfied	21 (55.3%)	17 (44.7%)	
Very satisfied	05 (13.2%)	07 (18.4%)	
Level of confidence in discharging patients			
Very Unconfident	01 (02.6%)	0	0.911
Unconfident	01 (02.6%)	0	
Neutral	04 (10.5%)	13 (34.2%)	
Confident	29 (76.3%)	19 (50.0%)	
Very confident	03 (07.9%)	06 (15.8%)	
Do you feel your discharge skills are time-consuming?			
Strongly disagree	02 (05.3%)	03 (07.9%)	0.622
Disagree	08 (21.1%)	06 (15.8%)	
Neutral	12 (31.6%)	17 (44.7%)	
Agree	12 (31.6%)	08 (21.1%)	
Strongly agree	04 (10.5%)	04 (10.5%)	
Having a discharge checklist would increase my confidence			
Strongly disagree	03 (07.9%)	0	0.324
Disagree	02 (05.3%)	04 (10.5%)	
Neutral	06 (15.8%)	14 (26.8%)	
Agree	15 (39.5%)	14 (36.8%)	
Strongly agree	12 (31.6%)	06 (15.8%)	

In Table 3, the differences in the levels of satisfaction, confidence, discharge skills, discharge checklist effects on confidence, and the frequency of discharge processes affecting patient safety between residents and other physicians did not reach statistical significance (all p>0.05).

**Table 3:** Comparison of satisfaction and confidence between physicians and residents before the discharge checklist was applied (n=38) *Others consist of specialists, fellows, and consultants only. ^§^P-values were calculated using Fisher’s exact test.

Satisfaction items	Resident, N (%) (n=27)	Others*, N (%) (n=11)	P-value^§^
Level of satisfaction with the discharge process			
Very dissatisfied	01 (03.7%)	01 (09.1%)	0.717
Dissatisfied	02 (07.4%)	0	
Neutral	05 (18.5%)	03 (27.3%)	
Satisfied	16 (59.3%)	05 (45.5%)	
Very satisfied	03 (11.1%)	02 (18.2%)	
Level of confidence in discharging patients			
Very unconfident	01 (03.7%)	0	0.446
Unconfident	01 (03.7%)	0	
Neutral	04 (14.8%)	0	
Confident	20 (74.1%)	09 (81.8%)	
Very confident	01 (03.7%)	02 (16.2%)	
Do you feel your discharge skills are time-consuming?			
Strongly disagree	02 (07.4%)	0	0.846
Disagree	06 (22.2%)	02 (18.2%)	
Neutral	08 (29.6%)	04 (36.4%)	
Agree	09 (33.3%)	03 (27.3%)	
Strongly agree	02 (07.4%)	02 (18.2%)	
Having a discharge checklist would increase my confidence			
Strongly disagree	02 (07.4%)	01 (09.1%)	0.38
Disagree	02 (07.4%)	0	
Neutral	06 (22.2%)	0	
Agree	09 (33.3%)	06 (54.5%)	
Strongly agree	08 (29.6%)	04 (36.4%)	
How often do you feel your discharge process might compromise patient safety			
Always	02 (07.4%)	01 (09.1%)	0.371
Often	03 (11.1%)	0	
Sometimes	07 (25.9%)	03 (27.3%)	
Rarely	10 (37.0%)	07 (63.6%)	
Never	05 (18.5%)	0	

When comparing the level of satisfaction and confidence between residents and other physicians after the discharge checklist was applied (Table 4), it was found that the resident groups were more likely to "strongly agree" that the discharge checklist would increase their confidence (p=0.010), whereas physicians were more likely to "agree" that their discharge skills are time-consuming (p=0.028).

**Table 4:** Comparison of satisfaction and confidence between physicians and residents after the discharge checklist was applied (n=38) ^§^P-values were calculated via Fisher’s exact test. *Others consist of only specialists, fellows, and consultants. **Significant at the p<0.05 level.

Satisfaction items	Resident, N (%) (n=27)	Others*, N (%) (n=11)	P-value^§^
Level of satisfaction with the discharge process			
Very dissatisfied	01 (03.7%)	0	0.533
Dissatisfied	0	01 (09.1%)	
Neutral	09 (33.3%)	03 (27.3%)	
Satisfied	11 (40.7%)	06 (54.5%)	
Very satisfied	06 (22.2%)	01 (09.1%)	
Level of confidence in discharging patients			
Very unconfident	0	0	0.146
Unconfident	0	0	
Neutral	10 (37.0%)	03 (27.3%)	
Confident	11 (40.7%)	08 (72.7%)	
Very confident	06 (22.2%)	0	
Do you feel your discharge skills are time-consuming?			
Strongly disagree	03 (11.1%)	0	0.028**
Disagree	05 (18.5%)	01 (09.1%)	
Neutral	13 (48.1%)	04 (36.4%)	
Agree	02 (07.4%)	06 (54.5%)	
Strongly agree	04 (14.8%)	0	
Having a discharge checklist would increase my confidence			
Strongly disagree	0	0	0.010**
Disagree	0	04 (36.4%)	
Neutral	11 (40.7%)	03 (27.3%)	
Agree	10 (37.0%)	04 (36.4%)	
Strongly agree	06 (22.2%)	0	
In your opinion, would you consider the information contained in the checklist to be comprehensive?			
Yes	23 (85.2%)	09 (81.8%)	1
No	04 (14.8%)	02 (18.2%)	

When measuring the relationship between physician satisfaction and the demographic data of the physicians (Table 5), it was revealed that physicians working in emergency medicine were more likely to be dissatisfied with the discharge checklist than non-emergency medicine physicians (p=0.049). No significant relationships were observed between satisfaction levels in terms of gender and professional rank (p>0.05).

**Table 5:** Relationships between demographic characteristics and satisfaction level after discharge (n=38) ^§^P-values were calculated using Fisher’s exact test. *Others include physicians, specialists, fellows, and consultants. **Significant at the p<0.05 level.

Factor	Satisfaction level		P-value^§^
	Dissatisfied, N (%) (n=14)	Satisfied, N (%) (n=24)	
Gender			
Male	12 (85.7%)	17 (70.8%)	0.438
Female	02 (14.3%)	07 (29.2%)	
Main specialty			
Emergency medicine	11 (78.6%)	11 (45.8%)	0.049**
Non-emergency medicine	03 (21.4%)	13 (54.2%)	
Professional rank			
Resident	10 (71.4%)	17 (70.8%)	1
Others*	04 (28.6%)	07 (29.2%)	

Table 6 shows that the confidence levels between males and females (p=0.456), between emergency medicine and non-emergency physicians (p=0.490), and between residents and other physicians (p=0.714) were not significantly different.

**Table 6:** Relationships between demographic characteristics and confidence level after the use of the discharge list (n=38) *Others include physicians, specialists, fellows, and consultants. ^§^P-values were calculated via Fisher’s exact test.

Factor	Confidence level		P-value^§^
	Not confident, N (%) (n=13)	Confident, N (%) (n=25)	
Gender			
Male	11 (84.6%)	18 (72.0%)	0.456
Female	02 (15.4%)	07 (28.0%)	
Main specialty			
Emergency medicine	09 (69.2%)	13 (52.0%)	0.49
Non-emergency medicine	04 (30.8%)	12 (48.0%)	
Professional rank			
Resident	10 (76.9%)	17 (68.0%)	0.714
Others*	03 (23.1%)	08 (32.0%)	

## Discussion

A critical phase in the continuity of care is patient discharge, when avoidable medical mistakes such as misunderstanding or insufficient information transmission can have a detrimental effect on patient outcomes after discharge, particularly readmission [13]. The aim of this study is to assess the impact of a discharge checklist on physicians' confidence, time management, and satisfaction in the ED. We evaluated the checklist's efficacy in enhancing the discharge process by comparing the satisfaction and confidence levels of physicians before and after its implementation. The study revealed no overall significant changes in physicians' satisfaction and confidence levels with the checklist's implementation, as discharge tasks remained time-consuming. However, residents reported increased confidence in their discharge skills, indicating the checklist's value as a training tool for less experienced physicians. On the other hand, there was a notable lack of satisfaction among ED physicians, suggesting that the checklist did not meet the demands of more experienced doctors in high-pressure settings.

In 2005, a prospective randomized controlled clinical trial revealed significant improvements in discharge planning involvement, health service access, confidence within discharge procedures, and the basis of previous experience for patients who received the discharge care plan [12]. A systematic review performed in 2017 revealed that multiple studies have tested the use of information technology (IT) in the discharge process. The results revealed that using IT, such as computer-generated and video-based discharge communication practices, is preferred by health care providers because it enhances patients’ understanding of medical instructions [18]. Additionally, a 2015 study in which a checklist was used improved physicians’ perceived confidence in discharge, reminded them to complete necessary discharge tasks, and increased the perceived efficiency of the discharge process [13].

On the other hand, this study revealed that there were no significant differences between the level of satisfaction and confidence before and after the use of the discharge checklist. Physicians tended to agree that discharge skills are time-consuming, whereas residents were more likely to agree that the discharge checklist increased their confidence levels. Another study reported that there was a slight improvement in each PGY's discharge skills before and after the discharge criterion was met [19]. However, younger emergency PGY residents tend to gain greater benefits and improvements following the implementation of discharge checklists than do senior residents. Interestingly, a well-designed discharge criterion implemented in the internal medicine department revealed that interns who were exposed to the criteria early in the academic curriculum had a higher reported frequency of completing key discharge tasks and similar confidence around discharge than did end-of-the-year interns. [20]

Limitations and recommendations

This study has several notable strengths, including its focused assessment of a discharge checklist and the use of a before-and-after study design, which allowed for systematic evaluation of changes in outcomes. However, there are several limitations that should be noted. As the study was only carried out at a single center, the findings cannot be generalized to other institutions or settings. Additionally, the study's short duration and comparatively small sample size may have affected the reliability of the findings. The use of self-reported data raises the possibility of bias since physicians' assessment might not accurately capture the checklist's true influence on discharge processes. To better understand the efficacy of the checklist and identify strategies to improve it, future research should consider qualitative methodologies and incorporate larger, more diverse samples from multiple centers.

## Conclusions

Physicians did not seem to improve satisfaction after the use of the discharge checklist. After the discharge checklist was used, physicians of higher rank expressed that their discharge skills were more time-consuming. More importantly, ED physicians' dissatisfaction with the discharge checklist was prevalent. In contrast, the residents believed that the discharge checklist increased their confidence after it was used. This study revealed that physician satisfaction remains the same despite the implementation of a new discharge checklist. Further studies with a larger cohort are recommended to provide a better understanding of the satisfaction and confidence of emergency physicians in the application of discharge checklists in our region.

## References

[1] Chugh A, Williams MV, Grigsby J, Coleman EA (2009). Better transitions: improving comprehension of discharge instructions. Front Health Serv Manage.

[2] Saidinejad M, Zorc J (2014). Mobile and web-based education: delivering emergency department discharge and aftercare instructions. Pediatr Emerg Care.

[3] Regalbuto R, Maurer MS, Chapel D, Mendez J, Shaffer JA (2014). Joint Commission requirements for discharge instructions in patients with heart failure: is understanding important for preventing readmissions?. J Card Fail.

[4] Dedhia P, Kravet S, Bulger J (2009). A quality improvement intervention to facilitate the transition of older adults from three hospitals back to their homes. J Am Geriatr Soc.

[5] D'Amore J, Murray J, Powers H, Johnson C (2011). Does telephone follow-up predict patient satisfaction and readmission?. Popul Health Manag.

[6] Ziaeian B, Araujo KL, Van Ness PH, Horwitz LI (2012). Medication reconciliation accuracy and patient understanding of intended medication changes on hospital discharge. J Gen Intern Med.

[7] Choi J (2013). Older adults' perceptions of pictograph-based discharge instructions after hip replacement surgery. J Gerontol Nurs.

[8] Coleman EA, Chugh A, Williams MV, Grigsby J, Glasheen JJ, McKenzie M, Min SJ (2013). Understanding and execution of discharge instructions. Am J Med Qual.

[9] Kripalani S, LeFevre F, Phillips CO, Williams MV, Basaviah P, Baker DW (2007). Deficits in communication and information transfer between hospital-based and primary care physicians: implications for patient safety and continuity of care. JAMA.

[10] Parkes J, Shepperd S (2000). Discharge planning from hospital to home. Cochrane Database Syst Rev.

[11] Bauer M, Fitzgerald L, Haesler E, Manfrin M (2009). Hospital discharge planning for frail older people and their family. Are we delivering best practice? A review of the evidence. J Clin Nurs.

[12] Preen DB, Bailey BE, Wright A (2005). Effects of a multidisciplinary, post-discharge continuance of care intervention on quality of life, discharge satisfaction, and hospital length of stay: a randomized controlled trial. Int J Qual Health Care.

[13] Garg T, Lee JY, Evans KH, Chen J, Shieh L (2015). Development and evaluation of an electronic health record-based best-practice discharge checklist for hospital patients. Jt Comm J Qual Patient Saf.

[14] Archbold RA, Laji K, Suliman A, Ranjadayalan K, Hemingway H, Timmis AD (1998). Evaluation of a computer-generated discharge summary for patients with acute coronary syndromes. Br J Gen Pract.

[15] Branger PJ, van der Wouden JC, Schudel BR, Verboog E, Duisterhout JS, van der Lei J, van Bemmel JH (1992). Electronic communication between providers of primary and secondary care. BMJ.

[16] O'Leary KJ, Liebovitz DM, Feinglass J (2009). Creating a better discharge summary: improvement in quality and timeliness using an electronic discharge summary. J Hosp Med.

[17] Waniga HM, Gerke T, Shoemaker A, Bourgoine D, Eamranond P (2016). The impact of revised discharge instructions on patient satisfaction. J Patient Exp.

[18] Newnham H, Barker A, Ritchie E, Hitchcock K, Gibbs H, Holton S (2017). Discharge communication practices and healthcare provider and patient preferences, satisfaction and comprehension: A systematic review. Int J Qual Health Care.

[19] Milano A, Stankewicz H, Stoltzfus J, Salen P (2019). The impact of a standardized checklist on transition of care during emergency department resident physician change of shift. West J Emerg Med.

[20] Eden EL, Rothenberger S, DeKosky A, Donovan AK (2022). The safe discharge checklist: a standardized discharge planning curriculum for medicine trainees. South Med J.

